# Draft genome sequence of *Bacillus velezensis* 2A-2B strain: a rhizospheric inhabitant of *Sporobolus airoides* (Torr.) Torr*.*, with antifungal activity against root rot causing phytopathogens

**DOI:** 10.1186/s40793-017-0289-4

**Published:** 2017-12-05

**Authors:** Inés Martínez-Raudales, Yumiko De La Cruz-Rodríguez, Alejandro Alvarado-Gutiérrez, Julio Vega-Arreguín, Ahuitz Fraire-Mayorga, Miguel Alvarado-Rodríguez, Victor Balderas-Hernández, Saúl Fraire-Velázquez

**Affiliations:** 1 Laboratorio Biología Integrativa de Plantas y Microorganismos, Unidad Académica de Ciencias Biológicas, Universidad Autónoma de Zacatecas, Av. Preparatoria s/n, Col. Agronómica, Zac. CP, -98067 Zacatecas, Mexico; 2 ENES-León, Universidad Nacional Autónoma de México, Mexico City, Mexico

**Keywords:** Chile wilt biocontrol, *Phytophthora capsici*, Fungal inhibition, Root rot biocontrol, Bacterial biocontrol agent, *Capsicum annuum* L

## Abstract

A *Bacillus velezensis* strain from the rhizosphere of *Sporobolus airoides* (Torr.) Torr*.*, a grass in central-north México, was isolated during a biocontrol of phytopathogens scrutiny study. The 2A-2B strain exhibited at least 60% of growth inhibition of virulent isolates of phytopathogens causing root rot. These phytopathogens include *Phytophthora capsici*, *Fusarium solani*, *Fusarium oxysporum* and *Rhizoctonia solani*. Furthermore, the 2A-2B strain is an indolacetic acid producer, and a plant inducer of PR1, which is an induced systemic resistance related gene in chili pepper plantlets. Whole genome sequencing was performed to generate a draft genome assembly of 3.953 MB with 46.36% of GC content, and a N50 of 294,737. The genome contains 3713 protein coding genes and 89 RNA genes. Moreover, comparative genome analysis revealed that the 2A-2B strain had the greatest identity (98.4%) with *Bacillus velezensis.*

## Introduction

Root rot causing microorganisms are among the most devastating phytopathogens of many horticultural crops resulting in considerable financial loss worldwide. Some of these pathogens include the oomycete *Phytophthora capsici*, and the fungi *Fusarium solani*, *Fusarium oxysporum* *,* and *Rhizoctonia solani*. Biocontrol strategies are important alternatives to keep some plant pathogens at low levels in affected crops, particularly when evaluating the risk of the use of pesticides on human health and the environment, and the social pressure to have innocuous horticultural food products. Biocontrol agents including bacterial strains that possess biocide activity against phytopathogens can also have the ability to invoke a systemic resistance (induced systemic resistance) in the host plant [1, 2]. In some cases, these bacterial strains are also able to promote the plant growth by inducing the biosynthesis of phytohormones [3, 4]. The rhizosphere is the area around the plant root that is inhabited by a unique population of microorganisms. The rhizospheric space is characterized by plant root exudates and usually by a high density and diversity of microorganisms. The root exudates have positive and negative effects in the interactions in the rhizosphere [5–7]. Colonizers of the rhizosphere are a great variety of microorganisms including bacteria that commonly have a friendly interaction with the plant host, suppressing at the same time some phytopathogens, and in some cases promoting plant growth [2, 7].

In seeking new options for biocontrol alternatives against phytopathogens, the genomic and biotechnological advances allows the deciphering of the molecular processes that regulate and induce the expression of many genes of plant-associated microorganisms. This will increase the possibilities for newer options of biocontrol agents with improved efficacy to deal with specific pathological problems of important crops.

In the present study we sampled soil and roots of cultivated and wild plants from Zacatecas state in the central-north region of Mexico, and isolated the bacterial strains from rhizosphere of *Sporobolus airoides* (Torr.) Torr*.* The bacteria reported in this study has the capacity to inhibit the growth of each of the four virulent isolates of the pathogens *P. capsici*, *R. solani*, *F. solani* and *F. oxysporum*, which are the causal agents of root rot in chili pepper crops. Levels of indoleacetic acid and biosynthesis of siderophores were analyzed, along with the induction of *NPR1*, a key gene controlling local resistance and systemic acquired resistance with multiple roles in plant immunity [8, 9]. In addition, the expression of the sesquiterpene cyclase gene involved in the isoprenoids pathway for the biosynthesis of phytoalexin capsidiol [10] was analyzed. The bacterial strain 2A-2B from this rhizosphere was selected for genome sequencing. A draft genome assembly of 3.953 MB was obtained and deposited in the NCBI GenBank (biosample SAMN05772828 and accession MLCV00000000), and in the Genomes OnLine Database (GOLD) with the accession Gp0177877.

## Organism information

### Classification and features

10.1601/nm.8505 strain 2A-2B is a Gram-positive bacterium, with rapid growth rate in LB liquid medium reaching the stationary phase after 13 h at 28 °C. In contrast, the growth rate of this strain was much slower in LB solid medium with the stationary phase attained at 24 h. The colonies in solid LB medium were observed as circular with pulvinated elevation and had filiform beige-opaque margins.

**Fig. 1:**
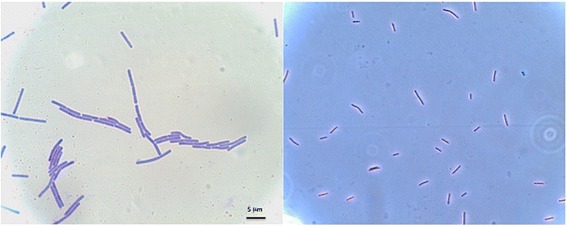
Cellular morphology of *B. velezensis* 2A-2B. Gram-positive bacteria under phase contrast with a Leica DM2500 compound microscope with Nomarski differential interference contrast

The strain was isolated from the rhizosphere of *Sporobolus airoides* (Torr.) Torr*.*, a wild grass growing in a grassland area of Morelos municipality in Zacatecas state, Mexico. This bacterium could grow well in mediums with high content of nutrients as LB, KB, TSA, PDA, and also in YMB medium. The optimal temperature for growth of this bacterium is 28 °C, although it was also capable of growing in temperatures of up to 35 °C. In root colonization assays in vitro in “mirasol” pepper plantlets (*Capsicum annuum* L. mirasol), no detrimental effect was seen in the plant. Furthermore, the 2A-2B 10.1601/nm.8505 strain produces indoleacetic acid (data not shown), a plant growth regulating auxin, but does not synthesize siderophores. The morphology of this motile rod shaped Gram-positive bacterium is shown in Fig. 1 and the general features in Table 1.

**Table 1:** Classification and general features of *Bacillus velezensis* strain 2A-2B [37]

MIGS ID	Property	Term	Evidence code^a^
	Classification	Domain: *Bacteria*	TAS [38]
		Phylum: *Firmicutes*	TAS [39, 40]
		Class: *Bacilli*	TAS [41]
		Order: *Bacillales*	TAS [42]
		Family: *Bacillaceae*	TAS [43]
		Genus: *Bacillus*	TAS [44]
		Species *Bacillus velezensis*	TAS [37]
		Strain: *2A-2B*	
	Gram stain	Positive	IDA
	Cell shape	Rod	IDA
	Motility	Motile	IDA
	Sporulation	Not specified	NAS
	Temperature range	20–35 °C	IDA
	Optimum temperature	28 °C	IDA
	pH range; Optimum	6.5–7.0; 6.5	IDA
	Carbon source	Heterotrophic	IDA
MIGS-6	Habitat	Soil	NAS
MIGS-6.3	Salinity	16% (w/v) NaCl	IDA
MIGS-22	Oxygen requirement	Aerobic	IDA
MIGS-15	Biotic relationship	Rhizosphere	NAS
MIGS-14	Pathogenicity	Non-pathogen	IDA
MIGS-4	Geographic location	México/Zacatecas, Morelos.	NAS
MIGS-5	Sample collection	2012	NAS
MIGS-4.1	Latitude	N 22° 49′ 12.354′´	NAS
MIGS-4.2	Longitude	W 102° 41′ 51.59299′´	NAS
MIGS-4.4	Altitude	2200.1 M	NAS

^a^Evidence codes - IDA: Inferred from Direct Assay; TAS: Traceable Author Statement (i.e., a direct report exists in the literature); NAS: Non-traceable Author Statement (i.e., not directly observed for the living, isolated sample, but based on a generally accepted property for the species, or anecdotal evidence). These evidence codes are from the Gene Ontology project [45]

**Fig. 2:**
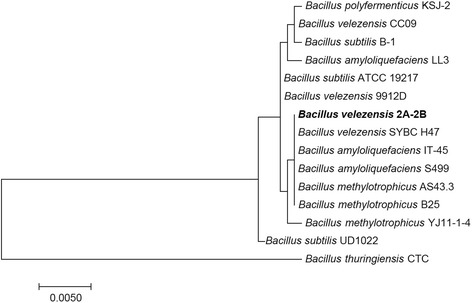
Phylogenetic tree with *B. velezensis* 2A-2B showing its relationships with species of the *Bacillus* genus. The tree was constructed based on 16S rRNA gene alignments in MEGA7 [11] applying the Neighbor-join method, and rooted using *Bacillus gibsoni* FJAT-10019 strain as an outgroup

The phylogenetic analysis based on 16S rRNA sequences using MEGA7 software [11] showed that 10.1601/nm.8505 2A-2B is evolutionarily positioned between 10.1601/nm.8505, 10.1601/nm.4868 and 10.1601/nm.20158 (Fig. 2). In recent studies of genome sequencing and comparative genomics of 10.1601/nm.8505 10.1601/strainfinder?urlappend=%3Fid%3DNRRL+B-41580, 10.1601/nm.20158 10.1601/strainfinder?urlappend=%3Fid%3DKACC+13015 and 10.1601/nm.22414 FZB42, it was established that these last two strains are heterotypic synonyms of 10.1601/nm.8505 [12], in this context and based in our results, the 2A-2B strain corresponds to the 10.1601/nm.8505 species.

**Fig. 3:**
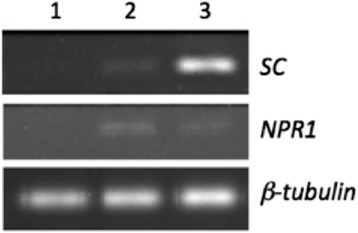
*NPR1* and Sesquiterpene cyclase genes induced in root inoculated plantlets of *Capsicum annuum* L*.* Genes level induction assessed by semi-quantitative RT-PCR. Assays with mixed total RNA from root and leaves of mirasol cultivar pepper plantlets in root inoculated with *B. velezensis 2A-2B* strain, at 12 hs post-inoculation. Lane 1, control plant not inoculated (mock); lane 2, plant in root inoculated with 2A-2B strain; lane 3, a *B. velezensis* strain inducer of *SC* gene in chili pepper. *SC*, sesquiterpene cyclase; *NPR1*, nonexpresser of PR genes

The 2A-2B strain of 10.1601/nm.8505 inoculated in roots of plantlets of *Capsicum annuum* L. mirasol cultivar induced expression of the sesquiterpene cyclase and *NPR1* genes (Fig. 3). Sesquiterpene cyclase is involved in the phytoalexin capsidiol biosynthesis pathway in *C. annuum* [10] and *NPR1* gene is a master regulator of systemic acquired resistance in response to biotic stress in plants [8, 13]. This work, in relation to the sampling of materials in the field and the activities performed in laboratory was done under national guidelines.

### Extended feature descriptions

**Fig. 4:**
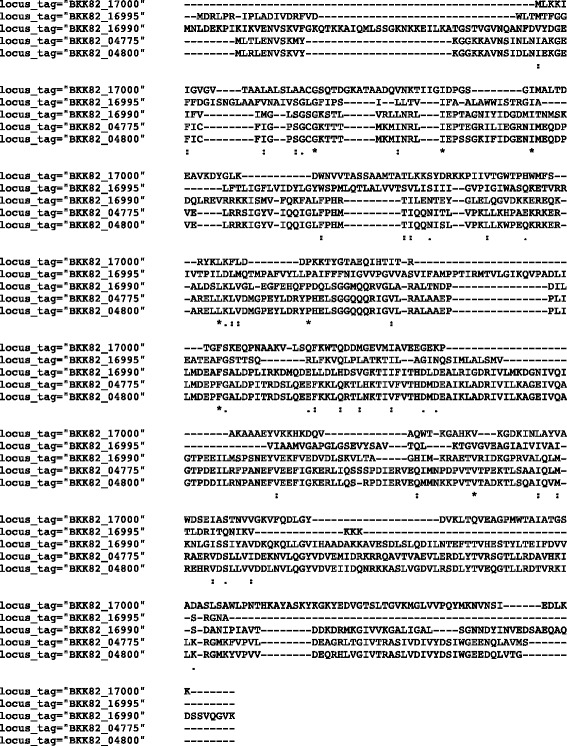
Alignment of glycine betaine/L-proline ABC transporter proteins present in the genome of *B. velezensis* 2A-2B strain. Semiconservative and nonconservative amino acids predominate

In physiological studies it is of relevance that this 10.1601/nm.8505 2A-2B strain is tolerant to salt. Tests of bacterial growth on solid medium with NaCl show that this bacterium is able to support up to 16% (*w*/*v*) (Table 1), whereas in other 10.1601/nm.4857 species and strains, the reported salt tolerance reach up to 12% [14]. This data motivated further studies on plant growth under saline soil condition with 2A-2B strain inoculations. In the genome of this strain five copies of the glycine betaine/L-proline ABC transporter ATP-binding protein are found, and their alignment with Clustal Omega software [15] shows both semiconservative and nonconservative amino acids (Fig. 4). Furthermore, it is found a glycine/betaine ABC transporter permease. This transport system is involved in osmoregulation by accumulating glycine betaine and other solutes under conditions of stress, with the functionality of osmoprotection [16, 17]. The fact that this bacterium contains two genes, one with 5 copies, related to the glycine betaine accumulation, suggests a possible role on the capacity of this microorganism in the osmoprotection mechanism under salt stress conditions.

## Genome sequencing information

### Genome project history

The 10.1601/nm.8505 2A-2B strain was selected due to its capacity to inhibit the growth of four pathogens, the causal agents of root rot in chili pepper. These include: *P. capsici*, *R. solani*, *F. solani*, and *F. oxysporum*. The 2A-2B strain was not pathogenic in “mirasol” chili pepper when inoculated in the roots.

The genome project of 10.1601/nm.8505 2A-2B was deposited in the GenBank NCBI database as a BioProject, Bio-sample and genome. The IDs for BioProject and genome accession numbers are PRJNA343056 and MLCV00000000, respectively. In the GOLD (genomes on line database) server, the assigned accession number is Gp0177877. A summary of the project information is shown in Table 2.

**Table 2:** Project information

MIGS ID	Property	Term
MIGS 31	Finishing quality	Quality draft
MIGS-28	Libraries used	illumina paired-end library
MIGS 29	Sequencing platforms	MiSeq illumina
MIGS 31.2	Fold coverage	38
MIGS 30	Assemblers	SPAdes Genome Assembler 3.8.1
MIGS 32	Gene calling method	NCBI Prokaryotic Genome Annotation Pipeline
	Locus Tag	BKK82
	Genbank ID	MLCV00000000
	GenBank Date of Release	2017–02-23
	GOLD ID	Gp0177877
	BIOPROJECT	PRJNA343056
MIGS 13	Source Material Identifier	2A-2B
	Project relevance	Biotechnological, Agricultural, Biocontrol

### Growth conditions and genomic DNA preparation

A sample taken from a colony of the 2A-2B strain was inoculated in 5 ml of LB liquid medium and cultured for 24 h at 150 rpm at 28 °C. The bacterial culture was centrifuged at 3500 g and the bacterial pellet was subjected to DNA extraction and purification based on the bacterial DNA isolation CTAB protocol [18]. The purity and concentration of the DNA was analyzed by agarose gel electrophoresis and in a Qubit 2.0 Fluorometer (Invitrogen). One nanogram of 10.1601/strainfinder?urlappend=%3Fid%3DDNA+in+5 ul of water was used to construct the genome libraries tagmented by PCR for Illumina sequencing. The quality and size of fragments in the libraries was verified in a Bioanalyzer (BioAnalizer 2010, Agilent Technologies). The libraries were subjected to standard normalization and 15 pM were used in the sequencing process.

### Genome sequencing and assembly

Purified genomic bacterial DNA was used to prepare libraries following Nextera Kit instructions (Illumina, San Diego Ca.). High-throughput sequencing was done under sequencing by synthesis protocol (MiSeq, Illumina) with a 2 × 75 paired-end run at the Sequencing Laboratory at the Unidad de Ciencias Biológicas, Universidad Autónoma de Zacatecas, México. SPAdes Genome Assembler 3.8.1 [19] was used to assemble the genome and the quality of the assembly was evaluated using QUAST 4.1 [20].

### Genome annotation

The protein-coding genes, structural RNAs and tRNAs in the draft genome were predicted using the NCBI Prokaryotic Genome Annotation Pipeline [21] and the GENIX Automated Bacterial Genome Annotation Pipeline [22].

Clusters of orthologous groups were predicted using the Web CD-search tool working with the COG database of orthologous protein families focusing on prokaryotes. Genes with Pfam domains and signal peptides were analyzed using the Pfam database [23], and the PrediSi software tool [24] respectively. Genes with transmembrane helices were analyzed with the CRISPRs web server [25]. A summary of all features of genome annotation is shown in Table 3.

**Table 3:** Genome statistics

Attribute	Value	% of Total
Genome size (bp)	3,958,607	100.0
DNA coding (bp)	3,545,146	89.55
DNA G + C (bp)	1,835,210	46.36
DNA scaffolds	64	100.0
Total genes	3891	100.0
Protein coding genes	3713	95.42
RNA genes	89	2.28
Pseudo genes	89	2.28
Genes in internal clusters	–	–
Genes with function prediction	3388	87.07
Genes assigned to COGs	3026	77,77
Genes with Pfam domains	1329	34.15
Genes with signal peptides	757	19.45
Genes with transmembrane helices	1049	26.95
CRISPR repeats	1	–

## Genome properties

**Fig. 5:**
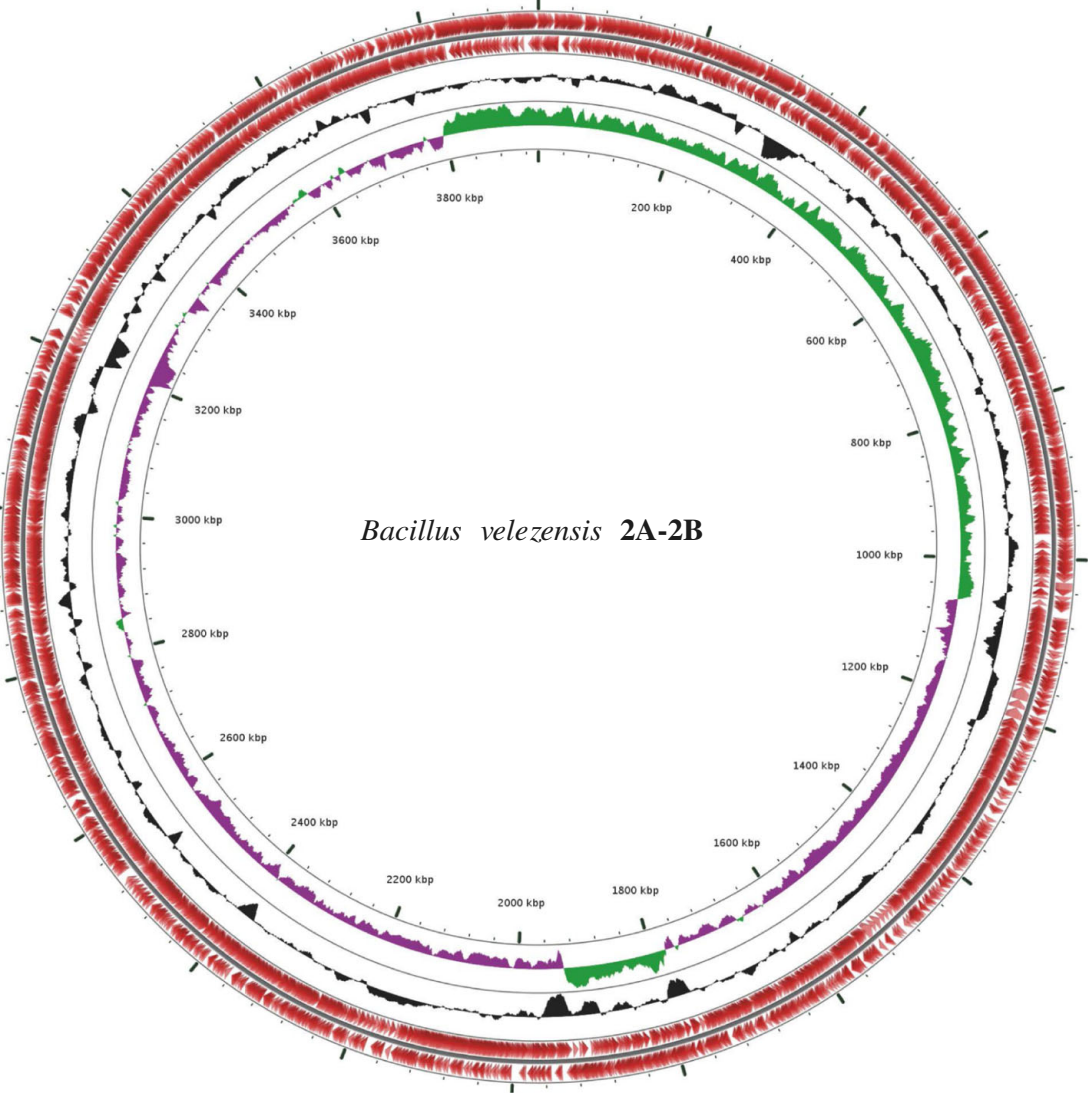
Circular map of the chromosome of *Bacillus velezensis* 2A-2B obtained with CGView comparative genomics tool [26]. From inner to outer rings: ring 1 scale marked in every 200 kbp, ring 2 GC skew (green +, purple -), ring 3 GC% content, ring 4 CDSs on reverse strand and ring 5 CDSs on forward strand

The draft genome contains 3,958,607 bp and a GC content of 46.36% with 3713 predicted protein-coding genes. Within this genome, 3388 genes had a predicted function, and 3026 were assigned to clusters of orthologous groups (COGs). In addition to the 3026 genes that were associated to general COG functional categories, 865 genes had not hit in COGs; this information is presented in Table 4, and the map of the genome generated with CGView comparative genomics tool [26] is represented in Fig. 5. Based on the results from sequence annotation and CD-search tool, we found 82 genes in the category of mobilome: prophages and transposons, suggesting that this 10.1601/nm.8505 2A-2B strain could eventually carry extrachromosomal elements when the prophage induction process takes place.

**Table 4:** Number of genes associated with general COG functional categories

Code	Value	%age	Description
J	221	5.60	Translation, ribosomal structure and biogenesis
A	0	0.0	RNA processing and modification
K	247	6.25	Transcription
L	112	2.84	Replication, recombination and repair
B	0	0.0	Chromatin structure and dynamics
D	63	1.60	Cell cycle control, Cell division, chromosome partitioning
V	78	1.98	Defense mechanisms
T	149	3.77	Signal transduction mechanisms
M	210	5.32	Cell wall/membrane biogenesis
N	61	1.54	Cell motility
U	28	0.71	Intracellular trafficking and secretion
O	112	2.84	Posttranslational modification, protein turnover, chaperones
Z	3	0.07	Cytoskeleton
C	167	4.23	Energy production and conversion
G	250	6.33	Carbohydrate transport and metabolism
E	279	7.07	Amino acid transport and metabolism
F	96	2.43	Nucleotide transport and metabolism
H	169	4.28	Coenzyme transport and metabolism
I	125	3.17	Lipid transport and metabolism
P	145	3.67	Inorganic ion transport and metabolism
Q	99	2.50	Secondary metabolites biosynthesis, transport and catabolism
X	82	2.07	Mobilome: prophages, transposons
R	173	4.38	General function prediction only
S	215	5.44	Function unknown
–	865	21.90	Not in COGs

## Insights from the genome sequence

Regarding the exhibited antifungal activity of this 10.1601/nm.8505 strain, we found genes in the genome that code for proteins of the *Bac* operon and the oligopeptide permease OppA. The Bac proteins are involved in the biosynthesis of bacilysin, a non-ribosomally synthesized dipeptide that is active against a range of bacteria and some fungi. The proteolysis of this dipeptide releases the non-proteinogenic amino acid L-anticapsin, which functions as a competitive inhibitor of glucosamine synthase and can result in the lysis of fungal cells [27, 28]. Also, a beta-glucanase and an endoglucanase are present in the genome of this bacterium. Similarly, surfactin synthetase gene which is present in the 2A-2B strain genome, adds to the capacity of this bacterium to contribute in the antifungal activity against the root rot causal agents. Furthermore, the surfactin lipopeptide of 10.1601/nm.10618 is well documented as elicitor of induced systemic resistance in plants [29–31]. In the genome of the 2A-2B strain of 10.1601/nm.8505, with a total of 3713 predicted-protein coding genes, the 1.98% corresponds to defense genes; and the 2.5% of genes corresponds to secondary metabolites biosynthesis. In these two functional categories of genes, a possible role in fungal inhibition may be important. In addition, the sesquiterpene cyclase and *NPR1* genes induced in chili pepper plantlets, during the 2A-2B strain root inoculation experiments, suggests that this lipopeptide is sensed by the signaling pathway in the plant’s defense system.

In other hand, in relation to the root bacterial colonization, the CheA and CheY genes are present in the genome of 2A-2B strain. These genes encode proteins that act as a two component system of bacterial chemotaxis, which is a response to chemical signals for controlling the direction of flagellar rotation [32–34]. With this two-component chemotaxis system and other plant exudate chemoreceptors, this bacterium could effectively reach the root tissue and proceeds with the plant tissue colonization.

In the carbohydrate transport and metabolism category, 250 genes (6.3% of total genes) were predicted in the 2A-2B strain genome including the PTS trehalose-specific enzyme IIBC component, xylose isomerase and a number of genes related to glucose metabolism. This suggests that the 2A-2B bacterium possesses a broad battery of genes coding for enzymes required to release a variety of carbon sources including some from plant exudates in the rhizosphere.

### Extended insights

The *B.velezensis* 2A-2B strain has a standard genome size compared to others 10.1601/nm.4857 species where the mean size fluctuate around 3.7 MB; the 2A-2B strain contains a genome of 3.96 MB. It is remarkable that the 2A-2B strain has only one 16S and one 23S rRNAs, whereas other 10.1601/nm.4857 species possesses seven, nine or ten of each.

**Fig. 6:**
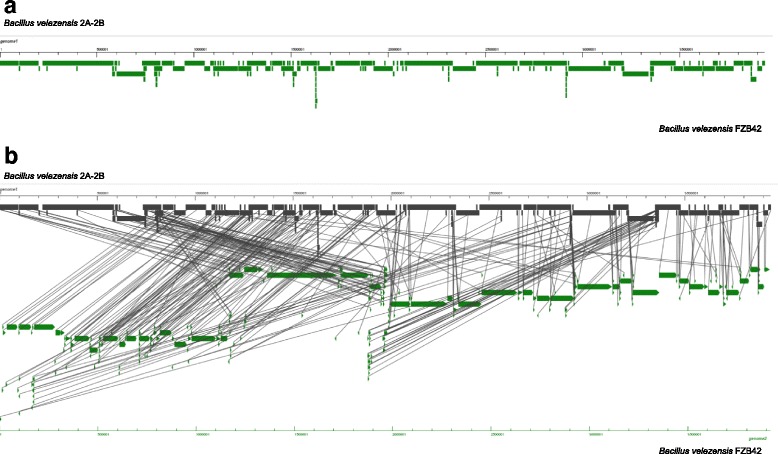
Synteny analysis between *B. velezensis* 2A-2B and *B. velezensis* FZB42 genomes. The *B. velezensis* FZB42 (acc: NC_009725.1) as reference genome. In **a**, synteny of 2A-2B strain vs. FZB42 strain. Blocks of synteny along the reference genome, and remarkable synteny with the sense DNA strand of 2A-2B strain is shown. In **b**, rearrangements of genomic segments in the 2A-2B strain in comparison to the genome of FZB42 strain. The synteny analysis was performed with VISTA software [35]

In genome comparison analysis to another accession of 10.1601/nm.8505 species, the alignment of 2A-2B and 10.1601/nm.8505 FZB42 (acc: NC_009725.1) as reference genome using VISTA software [35], shows blocks of synteny along the reference genome (Fig. 6A); in the genome of 2A-2B strain the great majority of synteny is found in the sense DNA strand, and a minor part in the antisense strand (Fig. 6B). The reference genome shows also fragments of synteny with multiple locations in the compared genome (Fig. 6A).

**Fig. 7:**
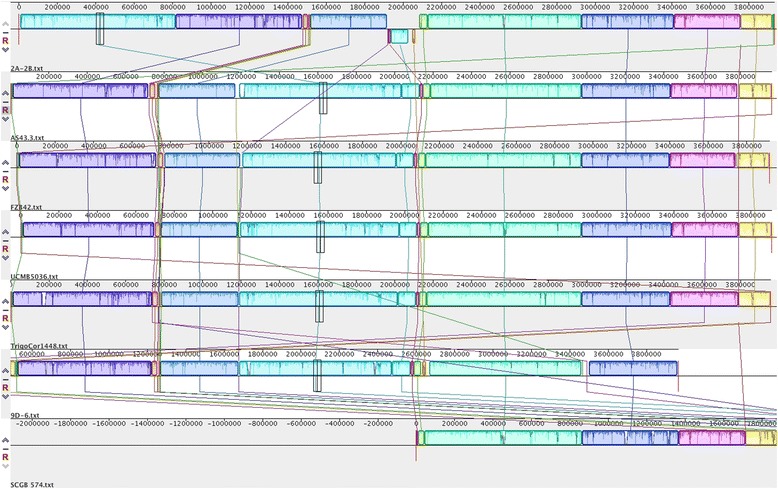
Multiple genome comparisons of 2A-2B and other 6 genomes, as a sample of the 89 *B. velezensis* genomes so far accessed in the NCBI GeneBank. In the 2A-2B *B. velezensis* strain genome 19 locally collinear blocks (LCBs) are found, this genome with two mayor rearrangements that imply the mayor LCB and a segment including three other LCBs in inverted orientation. The genome comparisons were performed with MAUVE software [36]

In multiple genome comparisons using MAUVE method, which is helpful in the localization of blocks of collinearity, rearrangements and inversions in conserved regions in genomes [36], in the 2A-2B strain of 10.1601/nm.8505 compared to other 6 genomes as a sample from the 89 10.1601/nm.8505 genomes so far accessed in the NCBI Gene Bank, 19 locally collinear blocks (LCBs) were found in the 2A-2B genome, 10 of mayor size, between 28,967 and 808,567 pb length, and 9 of small size, between 208 and 17,724 pb length. Four unique regions in the range of 1010 and 19,421 pb length localized outside of LCBs in the 2A-2B genome, without correspondence in the other 6 10.1601/nm.8505 genomes. Taking as reference any of the other 6 10.1601/nm.8505 genomes, the 6°, 7° and 8° LCBs in the 2A-2B genome are in inverted orientation. The 7° LCB in the 2A-2B genome is deleted in the SCGB574 10.1601/nm.8505 genome, and the 8° LCB of the 2A-2B genome is only conserved among the 2A-2B and 9D-6 of these 7 compared genomes. This alignment of 7 10.1601/nm.8505 genomes shows also that in the 2A-2B genome two events of large rearrangements have occurred, the greater LCB in 2A-2B genome is localized ahead of 4 LCBs in forward orientation compared to the other 6 genomes, and a segment that include three LCBs in the 2A-2B genome is in inverted orientation, in opposed situation in the other 6 compared genomes (Fig. 7).

## Conclusions

In this study, we obtained and characterized a draft genome of 10.1601/nm.8505, the 2A-2B strain isolated from *Sporobolus airoides* (Torr.) Torr. rhizosphere. The assembled genome contains a total of 3891 genes of which a high number of genes correspond to amino acid and carbohydrate transport and metabolism categories, and transcription and translation functions; whereas the 5.44% of genes have unknown functions. The antagonist characteristics of this bacterium against several fungal phytopathogens, could be explained in part in a number of described genes in the genome that are involved in the biosynthesis of compounds with toxic effects on fungal cell structures.

In comparative analysis with bacterial genomes in the NCBI DataBank using the draft genome and using the16S rRNA sequence, the 2A-2B strain was classified as 10.1601/nm.8505. The genome of the 2A-2B strain contains genes related to antifungal activity and systemic induced resistance in plants. A battery of genes is present that are involved in carbohydrate transport and metabolism, habilitating the bacterium for the assimilation of a diversity of carbon sources in the rhizosphere. Moreover, two genes, one with five copies, related to a transport system could be involved in the accumulation of glycine betaine with function of osmoprotection. Further analysis of the genome and specific genes of the 2A-2B strain will offer a better understanding of its biology, and the development of novel strategies in the biotechnological use of this bacterium.

## References

[1] Martinez-Hidalgo P, Garcia JM, Pozo MJ (2015). Induced systemic resistance against *Botrytis cinerea* by *Micromonospora* strains isolated from root nodules. Front Microbiol.

[2] Kang SM, Radhakrishnan R, Lee IJ (2015). *Bacillus amyloliquefaciens* subsp. *plantarum* GR53, a potent biocontrol agent resists Rhizoctonia disease on Chinese cabbage through hormonal and antioxidants regulation. World journal of microbiology & biotechnology.

[3] Couillerot O, Prigent-Combaret C, Caballero-Mellado J, Moenne-Loccoz Y (2009). *Pseudomonas fluorescens* and closely-related fluorescent pseudomonads as biocontrol agents of soil-borne phytopathogens. Lett Appl Microbiol.

[4] Dutkiewicz J, Mackiewicz B, Lemieszek MK, Golec M, Milanowski J (2016). *Pantoea agglomerans*: a mysterious bacterium of evil and good. Part IV. Beneficial effects. Annals of agricultural and environmental medicine : AAEM.

[5] Hinsinger P, Gobran GR, Gregory PJ, Wenzel WW (2005). Rhizosphere geometry and heterogeneity arising from root-mediated physical and chemical processes. The New phytologist.

[6] Badri DV, Vivanco JM (2009). Regulation and function of root exudates. Plant Cell Environ.

[7] York LM, Carminati A, Mooney SJ, Ritz K, Bennett MJ (2016). The holistic rhizosphere: integrating zones, processes, and semantics in the soil influenced by roots. J Exp Bot.

[8] Balderas-Hernandez VE, Alvarado-Rodriguez M, Fraire-Velazquez S (2013). Conserved versatile master regulators in signalling pathways in response to stress in plants. AoB PLANTS.

[9] Withers J, Dong X (2016). Posttranslational modifications of NPR1: a single protein playing multiple roles in plant immunity and physiology. PLoS Pathog.

[10] Back K, He S, Kim KU, Shin DH (1998). Cloning and bacterial expression of sesquiterpene cyclase, a key branch point enzyme for the synthesis of sesquiterpenoid phytoalexin capsidiol in UV-challenged leaves of Capsicum Annuum. Plant & cell physiology.

[11] Kumar S, Stecher G, Tamura K (2016). MEGA7: molecular evolutionary genetics analysis version 7.0 for bigger datasets. Mol Biol Evol.

[12] Dunlap C, Kim S, Kwon S, AP R (2016). *Bacillus velezensis* is not a later heterotypic synonym of *Bacillus amyloliquefaciens*; *Bacillus methylotrophicus*, *Bacillus amyloliquefaciens* subsp. plantarum and ‘*Bacillus oryzicola’* are later heterotypic synonyms of *Bacillus velezensis* based on phylogenomics. Int J Syst Evol Microbiol.

[13] Cao H, Glazebrook J, Clarke JD, Volko S, Dong X (1997). The *Arabidopsis* NPR1 gene that controls systemic acquired resistance encodes a novel protein containing ankyrin repeats. Cell.

[14] Niazi A, Manzoor S, Bejai S, Meijer J, Bongcam-Rudloff E (2014). Complete genome sequence of a plant associated bacterium *Bacillus amyloliquefaciens* subsp. plantarum UCMB5033. Stand Genomic Sci.

[15] Sievers F, Wilm A, Dineen D, Gibson TJ, Karplus K, Li W, Lopez R, McWilliam H, Remmert M, Soding J (2011). Fast, scalable generation of high-quality protein multiple sequence alignments using Clustal omega. Mol Syst Biol.

[16] Kunin CM, Tong HH, Miller DD, Abdel-Ghany Y, Poggi MC, LeRudulier D (1993). Effect of novel compound, 1-methyl-1-piperidino methane sulfonate (MPMS), on the osmoprotectant activity of glycine betaine, choline and L-proline in Escherichia Coli. Arch Microbiol.

[17] Kappes RM, Kempf B, Kneip S, Boch J, Gade J, Meier-Wagner J, Bremer E (1999). Two evolutionarily closely related ABC transporters mediate the uptake of choline for synthesis of the osmoprotectant glycine betaine in Bacillus Subtilis. Mol Microbiol.

[18] Wilson K: Preparation of genomic DNA from bacteria. *Current protocols in molecular biology* 2001, Chapter 2:Unit 2 4. 10.1002/0471142727.mb0204s56 18265184

[19] Bankevich A, Nurk S, Antipov D, Gurevich AA, Dvorkin M, Kulikov AS, Lesin VM, Nikolenko SI, Pham S, Prjibelski AD (2012). SPAdes: a new genome assembly algorithm and its applications to single-cell sequencing. Journal of computational biology: a journal of computational molecular cell biology.

[20] Gurevich A, Saveliev V, Vyahhi N, Tesler G (2013). QUAST: quality assessment tool for genome assemblies. Bioinformatics.

[21] Angiuoli SV, Gussman A, Klimke W, Cochrane G, Field D, Garrity G, Kodira CD, Kyrpides N, Madupu R, Markowitz V (2008). Toward an online repository of standard operating procedures (SOPs) for (meta)genomic annotation. Omics: a journal of Integr Biol.

[22] Kremer FS, Eslabao MR, Dellagostin OA, Pinto LD. Genix: a new online automated pipeline for bacterial genome annotation. FEMS Microbiol Lett. 2016;363(23) 10.1093/femsle/fnw263 27856568

[23] Finn RD, Coggill P, Eberhardt RY, Eddy SR, Mistry J, Mitchell AL, Potter SC, Punta M, Qureshi M, Sangrador-Vegas A (2016). The Pfam protein families database: towards a more sustainable future. Nucleic Acids Res.

[24] Hiller K, Grote A, Scheer M, Munch R, Jahn D: PrediSi: prediction of signal peptides and their cleavage positions. *Nucleic acids research* 2004, 32(Web Server issue):W375–W379. 10.1093/nar/gkh378 PMC441516 15215414

[25] Grissa I, Vergnaud G, Pourcel C: CRISPRFinder: a web tool to identify clustered regularly interspaced short palindromic repeats. *Nucleic acids research* 2007, 35(Web Server issue):W52–W57. 10.1093/nar/gkm360 PMC1933234 17537822

[26] Grant JR, Stothard P: The CGView Server: a comparative genomics tool for circular genomes. *Nucleic acids research* 2008, 36(Web Server issue):W181–W184. 10.1093/nar/gkn179 PMC2447734 18411202

[27] Koroglu TE, Ogulur I, Mutlu S, Yazgan-Karatas A, Ozcengiz G (2011). Global regulatory systems operating in bacilysin biosynthesis in *Bacillus subtilis*. J Mol Microbiol Biotechnol.

[28] Mariappan A, Makarewicz O, Chen XH, Borriss R (2012). Two-component response regulator DegU controls the expression of bacilysin in plant-growth-promoting bacterium *Bacillus amyloliquefaciens* FZB42. J Mol Microbiol Biotechnol.

[29] Liu H, Qu X, Gao L, Zhao S, Lu Z, Zhang C, Bie X (2016). Characterization of a *Bacillus subtilis* surfactin synthetase knockout and antimicrobial activity analysis. J Biotechnol.

[30] Jiang J, Gao L, Bie X, Lu Z, Liu H, Zhang C, Lu F, Zhao H (2016). Identification of novel surfactin derivatives from NRPS modification of Bacillus Subtilis and its antifungal activity against fusarium moniliforme. BMC Microbiol.

[31] Ongena M, Jourdan E, Adam A, Paquot M, Brans A, Joris B, Arpigny JL, Thonart P (2007). Surfactin and fengycin lipopeptides of *Bacillus subtilis* as elicitors of induced systemic resistance in plants. Environ Microbiol.

[32] Guhaniyogi J, Wu T, Patel SS, Stock AM (2008). Interaction of CheY with the C-terminal peptide of CheZ. J Bacteriol.

[33] Motaleb MA, Miller MR, Li C, Charon NW (2007). Phosphorylation assays of chemotaxis two-component system proteins in Borrelia burgdorferi. Methods Enzymol.

[34] Stock A, Chen T, Welsh D, Stock J (1988). CheA protein, a central regulator of bacterial chemotaxis, belongs to a family of proteins that control gene expression in response to changing environmental conditions. Proc Natl Acad Sci U S A.

[35] Poliakov A, Foong J, Brudno M, Dubchak I (2014). GenomeVISTA--an integrated software package for whole-genome alignment and visualization. Bioinformatics.

[36] Darling AC, Mau B, Blattner FR, Perna NT (2004). Mauve: multiple alignment of conserved genomic sequence with rearrangements. Genome Res.

[37] Ruiz-Garcia C, Bejar V, Martinez-Checa F, Llamas I, Quesada E (2005). *Bacillus velezensis* sp. nov., a surfactant-producing bacterium isolated from the river Velez in Malaga, southern Spain. Int J Syst Evol Microbiol.

[38] Woese CR, Fox GE (1977). Phylogenetic structure of the prokaryotic domain: the primary kingdoms. Proc Natl Acad Sci U S A.

[39] Gibbons N, Murray R (1978). Proposals concerning the higher taxa of bacteria. Int J Syst Bacteriol.

[40] Ludwig W., Karl-Heinz S.,B W. W: Revised road map to the phylum Firmicutes. In: *Bergey's Manual of Systematic Bacteriology: Volume 3: The Firmicutes.* Edited by Vos P, Boone DR, Garrity G, Castenholz RW, NR K, vol. 3: springer; 2001: 1–2.

[41] Ludwig W, Schleifer K-H, Whitman W: Class I. *Bacilli* class. nov. In: *Bergey's Manual of Systematics Bacteriology.* Edited by P. De Vos, G.M. Garrity, D. Jones, N.R. Krieg, W. Ludwig, F.A. Rainey, K.-H. Schleider, Whitman WB, vol. 3 (*The Firmicutes*), 2nd edn, edn. New York: Springer; 2015: 19–20.

[42] Prévot AR. Dictionnaire des Bactéries Pathogènes. In: Hauderoy P, Ehringer G, Guillot G, Magrou J, Prévot AR, Rosset D, Urbain A, editors. . 2nd ed. Masson, Paris: Masson et Cie; 1953. p. 1–692.

[43] Logan NAaV, P. D.: *Bacillaceae*. In: *Bergey's Manual of Systematics of Archaea and Bacteria.* vol. 1: John Wiley & Sons; 2015.

[44] Madhaiyan M, Poonguzhali S, Kwon SW, Sa TM (2010). Bacillus methylotrophicus sp. nov., a methanol-utilizing, plant-growth-promoting bacterium isolated from rice rhizosphere soil. Int J Syst Evol Microbiol.

[45] Ashburner M, Ball CA, Blake JA, Botstein D, Butler H, Cherry JM, Davis AP, Dolinski K, Dwight SS, Eppig JT (2000). Gene ontology: tool for the unification of biology. The gene ontology consortium. Nat Genet.

